# Neuronal Surface Antibody-Medicated Autoimmune Encephalitis (Limbic Encephalitis) in China: A Multiple-Center, Retrospective Study

**DOI:** 10.3389/fimmu.2021.621599

**Published:** 2021-02-17

**Authors:** Wei Shan, Huajun Yang, Qun Wang

**Affiliations:** ^1^Department of Neurology, Beijing Tiantan Hospital, Capital Medical University, Beijing, China; ^2^National Center for Clinical Medicine of Neurological Diseases, Beijing, China; ^3^Beijing Institute for Brain Disorders, Beijing, China

**Keywords:** encephalitis, demographic information, clinical symptoms, laboratory tests, relapse, treatment and outcome

## Abstract

**Objective:** The epidemiological characteristics of patients with antibody-medicated autoimmune encephalitis in China remain unclear, and a large-scale epidemiological survey is necessary.

**Methods:** A multiple-center retrospective study was performed. We collected 1,047 patients with suspected autoimmune encephalitis and ultimately enrolled 778 defined patients across centers in China. All patients were positive for serum [or cerebrospinal fluid (CSF)] antibodies. Demographic information and clinical data from January 2014 to January 2019 from 22 centers in China were reviewed.

**Results:** A total of 778 patients with autoimmune encephalitis were enrolled in the study. In general, the ratio of males to females was ~1.2:1. The main subtypes of autoimmune encephalitis were NMDAR-AE (61.35%), LGI-1-AE (20.61%), and GABAbR-AE (12.40%). According to the characteristics of age of onset, the incidence of autoimmune encephalitis showed a “double peak” distribution entailing a 20-year-old age group and a 60-year-old age group. We next analyzed the proportion of patients with tumors in this cohort. More specifically, there were 34 patients with tumors and 85 with tumor marker positivity. Relapse occurred in 81 patients within at least 1 year's follow up study: 52 with NMDAR-AE (18.2%); 19 with LGI-1-AE (16.8%); 5 with GABAbR-AE (9%); and 3 with CASPR2-AE.

**Interpretation:** Due to the vast differences in demographic features, the incidence of cancer and the genetic characteristics between the populations in China and Western countries, the demographics, sex distribution, concomitant tumor rate, clinical features, and relapse characteristics associated with autoimmune encephalitis in China shows a similar profile with Western countries with some minor differences.

## Introduction

Autoimmune encephalitis (AE) is associated with autoantibodies against neurosurface, synaptic or neuronal intracytoplasmic antigens. Since the first case of anti-N-methyl-D-aspartate receptor (NMDAR) encephalitis was identified (1), increasing numbers and subtypes of antibody-mediated encephalitis have been discovered, such as leucine-rich glioma-inactivated 1 (LGI-1), γ-aminobutyric acid type B receptor (GABAbR), and contactin-associated protein-2 (CASPR2) antibodies (2–4).

AE is induced when the immune system makes auto-antibodies that are not supposed to be there, these auto-antibodies begin to attack healthy brain cells wrongly identifying them as invade foreign though targeting special receptors in the brain during the brain inflammation (5). The antibodies can target the attack to receptors on the cell surface of healthy nerve cells in the brain or target the attack to synaptic receptors that are exposed to the attacking antibody or ion channels, during this process the healthy brain cells are destroyed or no longer function properly (6).

Several cohort studies have been described in Western countries, whereas a few systemic research and information have been reported in East Asia. Some centers have reported cases with neurological involvement in patients with autoimmune disease and information about treatment or outcomes (7–9). However, more detailed information from cohort studies regarding baseline information or whether neurological symptoms are attributable to secondary mechanisms is needed. Moreover, there are unanswered questions about differences in disease profiles regarding demographic information, genetic background, and cancer epidemiology between Eastern and Western regions.

Increased awareness and advanced testing methods in recent years have led to more frequent diagnoses of AE (10, 11). Nonetheless, the diverse clinical symptoms hamper accurate diagnosis and, consequently, appropriate treatment, thereby influencing outcomes, and prognoses for these patients.

Taking advantage of the National Center for Clinical Medicine of Neurological Diseases, a respective cohort of AE was established to describe the clinical characteristics, treatment regimens, and long-term outcomes of patients with this disease in China. We retrieved information from 22 hospital medical centers in China for this retrospective study. This paper aims to provide supporting evidence for the diagnosis and treatment of patients with AE based on the investigation of this cohort as well as the existing literature.

## Methods

### Standard Protocol Approvals, Registrations, and Patient Consent

This study was approved by the Ethics Committee of the Beijing Tiantan Hospital. The study was conducted in accordance with the Declaration of Helsinki, and all patients provided informed consent for the use of their medical records. All data analyzed in the study were strictly anonymous.

### Study Design and Population

Patients which suspected AE were enrolled consecutively from January 2014 to January 2019 at Beijing Tiantan Hospital, Tianjin Medical University Affiliated Hospital, Henan Province People Hospital, Nanjing Medical Hospital, Zheng Zhou Medical University Affiliated First Hospital, Qi Lu Hospital, Ningxia Medical School-Affiliated Hospital, Hebei Medical School Affiliated Hospital, Qingdao University Medical School Affiliated Hospital, Shanxi Province Hospital, and Baotou City Hospital, Xiangya Hosptial, Huaxi Hosptial, Jilin University affiliated First Hospital, Tongji Hospital, Zhongshan Hospital, Jilin University affiliated Third Hospital, The First Affiliated Hospital of Guangxi University, The first affiliated hospital, Sun Yat-sen University, Jilin University affiliated third hospital, and Wuhan Xiehe Hospital.

All the patients included in the study based on the following criteria: (1) Subacute onset (rapid progression of <3 months) of 1 or more of the 10 major groups of manifestations, including psychosis, memory deficit, speech disturbance, seizure, movement disorder, loss of consciousness, autonomic dysfunction, and central hypoventilation; (2) with or without CSF pleocytosis, encephalitis MRI features or EEG epileptic or slow-wave activity; (3) cerebrospinal fluid (CSF) or blood serum antibody testing positive for AE antibodies based on a cell-based assay (CBA) (FA 112d-1 for NMDAR, AMPAR, GABAbR, LGI-1, CASPR2; FA 1151 for LgLON5; Euroimmun Ag, Lubeck, Germany); (4) subtype cases more than 10 could be included and listed in this study; and (5) reasonable exclusion of other disorders.

Demographic data including age at onset, sex and ancillary test results including CSF or blood serum antibody test results, and magnetic resonance imaging (MRI) and electroencephalography (EEG) results were recorded. All patients were screened at least once for systemic tumors by computed tomography (CT), positron emission tomography (PET), MRI, or ultrasound at onset, if possible. Patients with tumors underwent tumor removal following the standard treatment for AE.

For the autoimmune encephalitis immunotherapy treatment included first-line [IV immunoglobulin (IVIG), corticosteroids, or plasmapheresis (PE) alone or combined] and second-line [rituximab (RTX) and cyclophosphamide (CTX) alone or combined] immunotherapies. Long-term immunotherapies [mycophenolate mofetil (MMF) or azathioprine (AZA) >1 year] and other immunotherapies [intrathecal methotrexate (MTX)] were also administered.

For the treatment effects evaluation, the modified Rankin Scale (mRS) was applied. For the relapse, it was defined as an exacerbation of previous symptoms or the occurrence of new symptoms after being stable for 2 months.

### Statistical Analysis

SPSS 22.0 was used for statistical analysis. Descriptive statistics were applied to analyze clinical data, such as medians and percentages. Figures were generated by GraphPad Prism 6.0. Quantitative data with normal distributions are presented as the mean ± SD; otherwise, medians with the interquartile range (IQR) are provided. A value of *p* < 0.05 was considered significant.

### Data Availability

Anonymized data not published within this article will be made available by request from the principal investigator, Qun Wang.

## Results

### Epidemiological Characteristics

**Figure 1:**
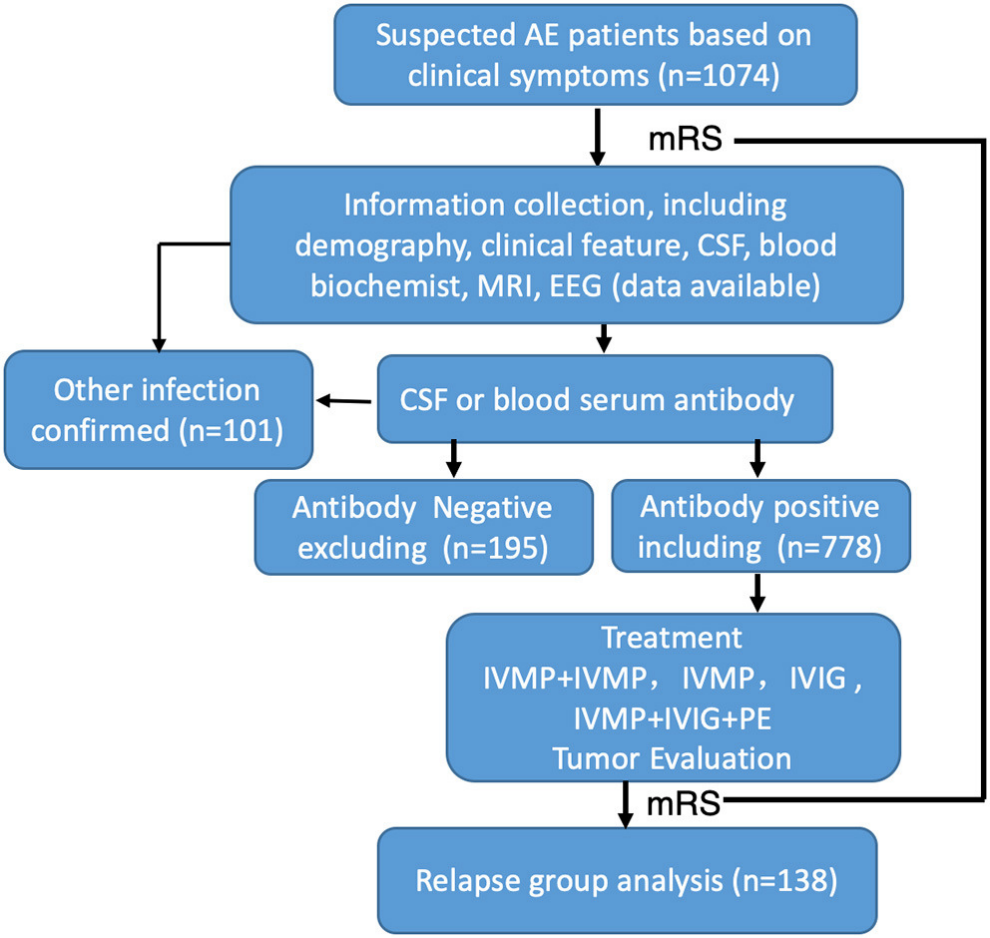
Flow chart of patients' inclusion and exclusion. One thousand and seventy-four patients were initially screened, 296 of them were excluded, a total of 778 AE patients were enrolled in the study.

A total of 1,047 suspected AE cases from 20 centers in China were collected for this study, and 778 defined patients were ultimately enrolled based on positive blood serum or CSF antibody assay results (Figure 1). In the collecting data set, only 74.31% patients reported antibody positive.

**Figure 2:**
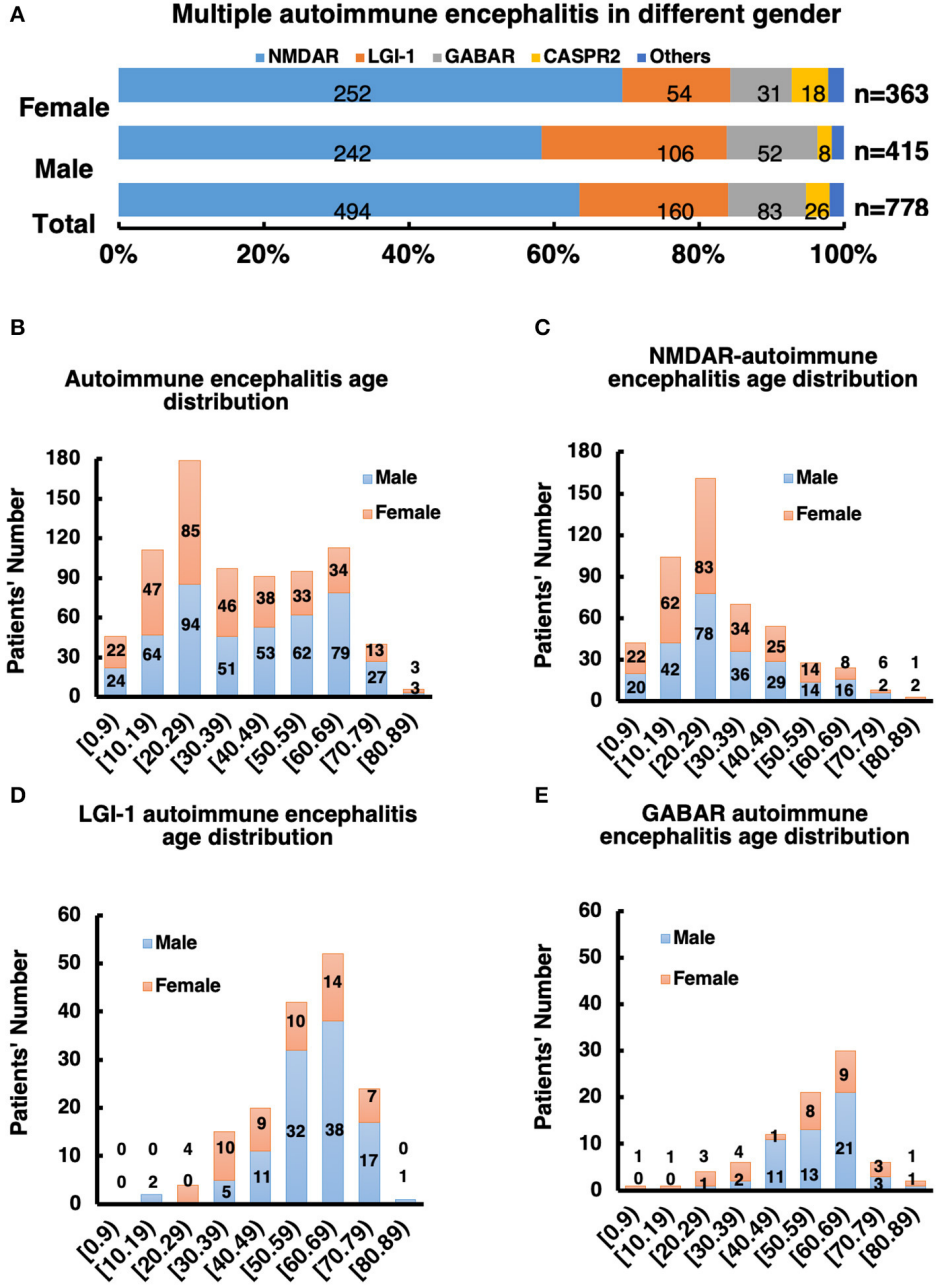
**(A)** Male to female ratio of all subtype AE patients. **(B–E)** Distribution of gender and age of AE patients and its subtype.

Among these autoimmune patients, the ratio of males to females was 1.2:1 (Figure 2A and Supplementary Tables 1, 2). The main subtypes of AE were NMDAR-AE (61.35%), LGI-1-AE (20.61%), and GABAbR-AE (12.40%) (Figure 2A and Supplementary Tables 1, 2). For each AE subtype, the sex ratio and age distribution of patients were also different. For the NMDAR-AE patients, the female ratio was 50.39% (Figure 2A and Supplementary Tables 1, 2), to compared with the data to Titulaer group's report, we also analysis the percentage of females in the age interval between 18 and 45 years old, the ratio is still 49.78%, this is different from reports in Western countries (80%). The median age in this autoimmune subtype was 27 years in females, ranging from 1 to 87 years, and 28 years in males, ranging from 2 to 83 years (Supplementary Tables 1, 2). For LGI-1-AE, the female ratio was ~29.69%, which was significantly lower than the male ratio. The age distribution in females was 58 years, ranging from 23 to 78 years, and the median age in males was 61 years, ranging from 15 to 82 years. Among GABAbR-AE patients, the female ratio was ~35.06%, also significantly lower than the male ratio. The median age in females and males was 56 and 57 years, ranging from 17 to 84 years and from 28 to 82 years, respectively (Supplementary Tables 1, 2).

Patients under 18 years of age comprise a special group that needs intensive care in the hospital, and we analyzed ratios across subgroups in this study. The patient number and percentage in the NMDAR subgroup were significantly higher than those in the other groups: the total NMDAR subgroup patient number was 77, and ~25.71% were under 18 years old (*p* > 0.01); in the other groups, the percentages were 1.25% (LGI-1), 2.41% (GABAbR), and 15.38% (CRASPR).

The distribution of AE subtypes varied between the sexes. In the male group, 55.59% had NMDAR-AE vs. 68.33% in the female group, and 26.47% had LGI-1 vs. 13.52%; 14.41% of males had GABAbR-AE vs. 9.96% of females, and 2.05% of males had CASPR2-AE vs. 5.69% of females (Figures 2B–E).

According to the characteristics of age of onset, the incidence of AE exhibited a “double peak” distribution, with a 20-year-old age group and a 60-year-old age group (Figure 2B). The age of onset of NMDAR-AE was mainly ~20 years old (Figure 2C), whereas that of LGI-1 and GABAbR-AE was mainly at ~60 years old (Figures 2D,E), which constituted a later peak.

### Clinical Characteristics

Based on our study, the most common clinical manifestations of AE in the initial stage were psychosis (341 patients, 54.91%) and seizure (397 patients, 63.93%). In this study, we also noted that headache (34.65%) and fever (33.60%) were more frequent (*p* < 0.001) in the NMDAR subgroup than in the other subgroups, with rates of <8%. The percentage of patients with a decrease in consciousness was lower in the LGI-1-AE subgroup, at only 7.03% of patients, than in the other groups, which was over 20% (*p* < 0.001). However, memory deficits were more likely in the LGI-1-AE subgroup, at ~62.5%, which was significantly higher than that in the other subgroups (*p* < 0.001). In the GABAbR-AE subgroup, movement disorders were a rare symptom that appeared in ~3.90% of patients. More detailed information about these symptoms are provided in Table 1.

**Table 1:** On-set clinical symptom of patients with autoimmune encephalitis.

**On-set**	**All**	**NMDAR-AE**	**LGI-1 AE**	**GABAbR-AE**	**CRASPR-AE**
	**(*n* = 778)**	**(*n* = 494)**	**(*n* = 160)**	**(*n* = 83)**	**Others (41)**
Psychosis	414 (53.21%)	270 (54.66%)	79 (49.38%)	42 (50.60%)	23 (56.10%)
Seizures	482 (61.95)	279 (56.48%)	120 (75.00%)	70 (84.34%)	13 (31.71)
Fever	181 (23.26%)	164 (33.20%)	3 (1.88%)	6 (7.23%)	8 (19.51%)
Consciousness-decrease	200 (25.71%)	141 (28.54%)	16 (10.00%)	35 (42.17%)	8 (19.51%)
Memory deficit	177 (22.75%)	47 (9.51%)	97 (60.63%)	24 (28.92%)	9 (21.95%)
Speech disturbance	126 (16.19%)	89 (18.02%)	14 (8.75%)	14 (16.87%)	9 (21.95%)
Movement disorder	111 (14.27%)	82 (16.60%)	14 (8.75%)	4 (4.82%)	11 (26.83%)
Sleep disorder	94 (12.08%)	58 (11.74%)	18 (11.25)	13 (15.66%)	5 (12.20%)
Headache	175 (22.49%)	161 (32.59%)	7 (4.38%)	5 (6.02%)	2 (4.88%)
Visual defect	42 (5.40%)	35 (7.09%)	3 (1.88%)	1 (1.20%)	3 (7.32%)

*LGI-1 seizure included FBDS, 49 patients (40.83%)*.

Associated tumors are detected in AE patients, and oncologic management (chemotherapy or tumor resection) is essential for improvement. In this study, tumors were diagnosed in 34 (8.97%) of 379 patients who underwent tumor screening tests, including thyroid ultrasound, abdominal ultrasound, gynecological ultrasound, breast ultrasound, thoracoabdominal CT, and whole-body PET/CT. Of 212 anti-NMDAR-AE patients, 23 were diagnosed with tumors (10.85%). Of the 19 females, 16 (84.2%) had ovarian teratomas, two had hamartoma, and one had small-cell lung cancer; of the 4 males, 3 had small-cell lung cancer and one had gallbladder small-cell carcinoma. All female patients with ovarian teratomas underwent tumor removal, whereas the patient with lung cancer was treated with palliative therapy by an internist and later died of cancer. In the GABAbR-AE group, a total of 9 patients were diagnosed with cancer, including 8 with small-cell lung cancer and one with a mediastinal tumor. To our surprise, LGI-1-AE rarely correlated with cancer in follow-up studies (Supplementary Table 4).

### Auxiliary Examinations

In this study, ~510 patients underwent brain MRI at onset, and 295 (61.2%) had abnormal fluid-attenuated inversion recovery (FLAIR) sequence signals, including 45.45% (115/253 patients) with NMDAR-AE, 82.14% (115/140 patients) with LGI-1-AE, 75.31% (61/81 patients) with GABAbR-AE, and 91.67% (33/36 patients) with CASPR-AE and other types. MRI findings were mainly abnormal in the medial temporal lobe, including in ~36.44% of the patients with NMDAR-AE, 46.08% with LGI-1-AE, 50.00% with GABAbR-AE, and 61.54% with CASPR-AE and other types. Other involved areas included the frontal, parietal, and occipital cortices, the diencephalon, the cerebellum, and the brainstem.

Abnormal EEG findings were obtained in 386 of 525 patients (73.52%): 351 (66.85%) had slow activity, and 32 (6.10%) showed epileptiform discharges. For each subtype of AE, abnormal EEG percentages were 79.16% (247/312) in those with NMDAR-AE, 61.29% (76/126) in those with LGI-1-AE, 63.79% (37/58) in those with GABAbR-AE, and 89.66% (26/29) in those with CASPR-AE and other types (Supplementary Table 4).

In total, ~597 patients were tested for CSF white blood cells in the initial stage, with levels of 39.26 (count per mm^3^) in NMDAR-AE patients, 7.79 in LGI-1-AE patients, and 29.93 in GABAbR-AE patients. Moreover, protein levels in the CSF at onset were elevated in 394 patients, including 0.47 mg/dL in NMDAR-AE patients, 0.553 mg/dL in LGI-1-AE patients, and 0.64 mg/dL in GABAbR-AE patients (Supplementary Table 3).

### Treatment Outcomes

**Figure 3:**
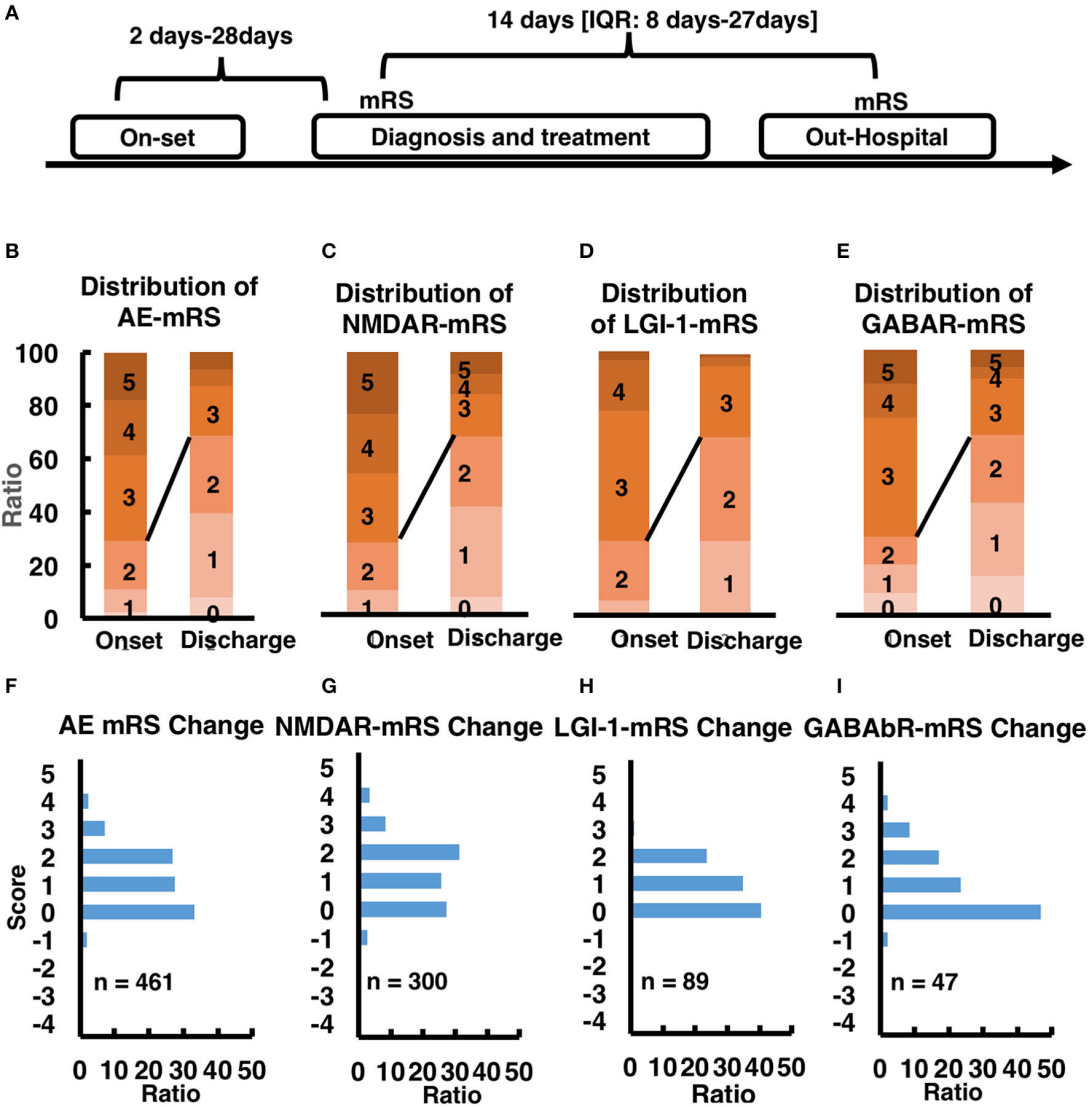
**(A)** time line of mRS-taking. **(B–E)** mRS score at onset stage and out-hospital stage in AE patients and other subtype AE patients. **(F–I)** mRS score change after treatment in AE patients and other subtype AE patients.

There are various treatment approaches for AE patients, such as first-line corticosteroid treatment, intravenous immunoglobulin treatment, plasma exchange treatment and second-line rituximab and cyclophosphamide treatment. Overall, 768/778 (98.76%) patients received first-line immunotherapy in hospital, a combined regimen of repeated steroids and IVIG in most cases (444 patients, 57.1%). A total of 665 (85.48%) patients received steroids, of whom 221 (33.23%) received pulsed IV methylprednisolone. IVIG was administered to 553 (71.08%) patients, and 20 (2.5%) patients underwent PE. Conversely, second-line immunotherapy was administered to only a small proportion of the patients. Thirty-six (4.5%) patients received RTX or CTX. Our statistical analysis, especially for the in-hospital patients, all the patients from on-set to diagnosis and treatment taking about 2–28 days, for the in-hospital treatment 14 days (IQR: 8–27 days). During this treatment period, we evaluated the patient's condition and the effect of treatment with the Modified Rankin Scale (mRS), and the whole process showed in Figure 3A. In general, 75% of the patients (*n* = 461) improved after treatment, the change of mRS distribution before and after treatment could be found in Figure 3B (mRS distribution before and after treatment) and Figure 3F (mRS change distribution ratio mapping), for more detailed information about other subtypes such as NMDAR, LGI-1 and GABAbR could be found in Figures 3C–E,G–I. Generally, the treatment-response NMDAR-AE is better than LGI-1 AE, LGI-1 AE is better than GABAbR-AE. However, due to lacking the mRS recording information for the following-up study, we could not provide more information about mRS evaluation in this study.

### Relapse Group

For more than 1 year' follow up study, a total of 138 relapsed patients (17.74%) were evaluated. The median duration from onset to the first relapse was 192 days (IQR: 100–354). Of the relapsed cases, 51 patients (36.96%) were female, 36 patients (21.83%) were under 18 years old, 89 patients (64.49%) had NMDAR-AE, 31 patients (22.46%) had LGI-1-AE, 9 patients (10.11%) had GABAbR-AE, 5 patients (3.62%) had CASPR-AE, and 4 patients (2.90%) had other types. Regarding the relationship between relapse and cancer, two patients with NMDAR-AE relapse (2/89) had ovarian teratomas at disease onset, and 4 patients with GABAbR-AE relapse (4/9) had lung cancer at disease onset.

## Discussion

In this study, we showed that our collected and analyzed data are comparable with those in previous literature (Table 2), and we provided additional detailed information. The incidence of antibody-mediated AE is increasing over time, and an increasing number of patients are identified after being diagnosed with other diseases, especially psychiatric disorders. Overall, it is of great importance to be aware of antibody-mediated AE in patients with various symptoms, which will lead to an increased amount of testing for autoantibodies and, consequently, an increased number of patients diagnosed with this disease.

**Table 2:** Comparison of several representative cohort or case series of antibody-mediated autoimmune encephalitis in China and western countries.

	**#of patients (region, centers)**	**Demographic information**	**Common types**	**Clinical characteristics**	**Auxiliary examination**	**Treatment**	**Prognosis and outcome**
This study	*N* = 1,027 *N* = 778 (China, 23 centers)	1–87 y.o. (median = 35); female 45.50%	Anti-NMDAR (61.35%), Anti-LGi1 (20.57%), Anti-GABAbR (10.67%), others (5.27%)	Psychosis (53.21%), seizures (61.95%), headache (32.59%), fever (33.20%), consciousness impairment (28.54%)	MRI: abnormal FLAIR signals (61.2%) mainly in the medial temporal lobe. EEG: slow activity (61.67%) and epileptiform discharges (6.88%)	First-line: steroid (85.48%) and IVIG (66.77%), mostly combined (57.1%); PE (2.5%) Second-line: RTX or CTX (4.5%)	17.74% with relapse: anti-NMDAR (64.49%), anti-LGi1 (22.46%), anti-GABAR (10.11%), anti-Caspr2 (3.62%)
Deng et al.	*N* = 86 (China, single-center)	Mean = 32.9 y.o.; female 44.19%	Anti-NMDAR (83.72%), Anti-LGi1 (4.65%), Anti-GABAbR (5.81%), anti-Caspr2 (3.49%), others (3.49%)	Psychiatric disturbance, epilepsy, autonomic dysfunction, sleep disorders, consciousness disorders	MRI: abnormal in 50%, mainly insular/hippocampal abnormality. EEG: abnormal in 71%; typically slow waves and sharp waves, mainly in the frontal area (48.8%) and area centralis (32.6%)	steroid or in combination with PE, IVIG, or immunosuppressive agents like cyclophosphamide	Immune therapy administered within 15 days from onset was associated with a higher rate of mRS score difference ≥ 2
Gu et al.	*N* = 189 (Southwest China, multi-center)	1–70 y.o. (median = 16); female 61.38%	Anti-NMDAR (80.95%), Anti-LGi1 (4.76%), Anti-GABAbR (7.41%), anti-Caspr2 (2.65%), others (4.23%)	N/A in detail; 62.22% of adult and 10.10% of children admitted to ICU	N/A	N/A	76.71% of male patients and 92.92% of female patients had a good prognosis
Probasco et al.	*N* = 61 (Sweden, single-center)	Median = 54 y.o.; female 54%	Anti-NMDAR (13.11%), Anti-LGi1 (8.20%), Anti-VKGC (6.56%), anti-GAD65 (6.56%), other seropositive (18.03%)	Short-term memory impairment (75%), cerebellar signs (77%), focal weakness (61%), focal numbness (57%), movement disorder (64%), seizures (41%)	FDG-PET/CT: brain regional abnormal metabolism 85%	N/A	N/A
Titulaer et al.	*N* = 577 (USA, multi-center)	<18 y.o.: 37%; >18 y.o.:63%; female 81%	Anti-NMDAR specifically	Behavior problem (65%), movement disorder (50%), Seizures (50%); 77% admitted to ICU	MRI: abnormal in 33% EEG: abnormal in 90%	First-line immunotherapy: 92% Second-line immunotherapy: 27% Tumor removal y: 2%	Favorable outcomes: 81% Relapse: 11%
Mueller et al.	*N* = 150 (Germany, multicenter)	Anti-NMDAR: mean = 30.3 y.o. Anti-LGi1: mean = 62.7 y.o.	Anti-NMDAR and anti-Lgi1 specifically	Anti-NMDAR: seizures (73%), psychiatric symptoms (92%), movement disorder (45%), autonomic dysfunction (39%) Anti-Lgi1: seizures (76%), psychiatric symptoms (57%), movement disorder (11%), autonomic dysfunction (12%)	Anti-NMDAR: abnormal MRI in 57%, abnormal EEG in 75% Anti-LGi1: abnormal MRI in 74%, abnormal EEG in 78%	N/A	N/A
Xu et al. (12)	*N* = 220 (China single-center)	<18 y.o.: 31.08%; >18 y.o.: 68.92%, female 81%	Anti-NMDAR specifically	Psychosis 82.7%, seizures 80.9%, Fever 57.3%, decrease of consciousness 53.2%, 30.9% admitted to ICU	MRI: abnormal in 35.9% EEG: abnormal in 51.4%	First-line immunotherapy: 99.5% Second-line immunotherapy: 7.3%	94.1% improvement 3.6% stable 2.3% died
Guan et al. (13)	*N* = 531 (China single-center)	N/A.	Anti-NMDAR (79.7%), Anti-LGi1 (12.8%), Anti-GABAbR (5.6%), others (1.8%)	N/A.	N/A.	N/A.	Relapse: 23.5%

To our knowledge, this is the largest Chinese AE cohort to date. We identified anti-NMDAR encephalitis, anti-LGI-1 encephalitis, anti-GABAbR encephalitis, and anti-CASPR encephalitis as the most common subtypes in our cohort, which is similar to previous studies in China and in Western countries (7, 8, 14, 15).

The mode of onset in antibody-mediated AE is mostly acute or subacute. The common clinical manifestations reported include psychiatric disorders, epileptic seizures, involuntary movement, and sleep disorders (16). Our results are similar to previous studies. In our study, the most common clinical symptoms in patients were psychiatric symptoms and epileptic seizures, consistent with other Chinese cohorts and most Western cohorts (7, 8, 14, 15). In addition, consciousness impairment, memory deficit, speech disturbance, movement disorder and sleep disorder are common symptoms worth noting (17). In this study, we observed that headache and fever were more frequent in anti-NMDAR AE than in other subtypes. Scholars have speculated that viruses can trigger a pro-inflammatory state that activates the immune system, including microglia and immune cells in the central nervous system. Over activated immune cells produce an autoimmune response against the CNS (18). In previous studies, HSV has been found to be an important inducer of anti-NMDAR AE (19). In addition to HSV, other viral antigens, such as HIV, rubella virus, cytomegalovirus and Epstein-Barr virus, may also be positive in some patients (20–23). This might explain the higher prevalence of fever and headache in anti-NMDAR AE, as such symptoms are relevant to viral infection.

In our study, there were 34 patients with tumors and 85 patients diagnosed with abnormal tumor markers [including Alpha fetoprotein (AFP), CA125, CA242, CA72-4, CA50, CA19-9, carcinoembryonic antigen (CEA), Prostate specific antigen (t-PSA), Cytokeratin-19-fragment (CYFYA21-1), neuron specific enolase (NSE), Squamous cell carcinoma antigen (SCC), pro-gastrin-releasing peptide (ProGRP)]. In the case of anti-NMDAR AE, the prevalence of an underlying neoplasm varies among studies. For example, Titulaer et al. reported that 38% of patients had a tumor and that Asian patients were more likely (45%) to have a teratoma (15). However, only 8.39% of the patients in our cohort had a tumor, with ovarian teratoma comprising 84.2% of the tumors diagnosed in females. Other studies of Chinese or Asian cohorts have also reported a relatively low prevalence of tumors (Supplementary Table 4) [Lim et al. (24), 22.7%; Wang et al. (25), 8%; Liu et al. (22), 6.7%; Zhang et al. (26), 8.1%]. The heterogeneity among Asian and Western populations might be due to different genetic backgrounds and epidemiologic factors. In addition, sample size differences and selection bias are also factors that might influence the results. Thus, future studies are required to confirm the association between AE and tumors.

Brain MRI and EEG are the main auxiliary examinations significant for the diagnosis of antibody-mediated AE. Findings on brain MRI and EEG analyses provide evidence that AE is a “diffuse encephalopathy” (7, 15, 27). In this study, abnormal signals on brain MRI were detected in 61.2% of the patients, predominantly in the medial temporal lobe. This result is similar to another study in southwestern China (abnormal in 50%, mainly insular/hippocampal abnormality) (8). This result might explain the high incidence of epileptic seizures in patients with AE, as medial temporal structures, including the hippocampus, are critical in the neural circuits that play an important role in seizure propagation (28, 29). Abnormal signals in other areas of the cortex, diencephalon, brainstem, and cerebellum have also been reported (27). Abnormal EEG signals were more commonly observed in this study and in previous studies. Most of the EEG abnormalities were slow waves, which are indicative of brain structural lesions. Epileptiform discharges such as sharp waves were also observed in the present and previous studies, indicating clinical or subclinical seizure activity (7, 14, 15).

In the management of antibody-mediated AE, repeated first-line immunotherapy, mostly a combination of steroids and IVIG, was most frequently used in our cohort. In contrast, second-line immunotherapy was applied in a relatively small portion of patients due to RTX's off-label use for AE in China (7, 15). This situation is similar to previous reports in China, where the second-line immunotherapy is limited by RTX's off-label use for AE, cost, hospitalization requirements, and concerns about side effects (7, 8, 30). While in previous reports in Western countries, the second-line immunotherapy was applied in a much larger proportion of patients because it's commonly used in those who failed the first-line therapy (15). The proportion of patients who received improvements and favorable outcomes was relatively high, which are similar between reports from China and Western countries (8, 15, 30). Moreover, Titulaer et al. suggested that predictors of good outcome were early treatment and lack of ICU admission, and predictors of good outcome and the magnitude of effect of second-line immunotherapy were comparable to the entire cohort, validating the therapeutic significance of second-line immunotherapy which is not fully utilized yet in China (15). Relapses (clinical symptoms descriptions) were relatively common in our cohort and other reports [17.3% (30, 31)] in China, which is slightly higher than Western reports [12% (15)]. This might be due to genetic differences among different human races. The serum or CSF antibody titer did not consistent with clinical severity. In some relapse cases, antibodies could only be detected in the CSF, as previously reported (7, 8, 15). This suggests that we should combine clinical manifestations and antibody titer in disease monitoring in clinical practice.

The main limitations of this study as the same as other retrospective researches. The patients were recruited from the clearly diagnosed autoimmune encephalitis, which excluded the other unclearly antibody medicated autoimmune encephalitis, not included all potential autoimmune encephalitis patients (antibody test negative group). Even though the diagnosis of autoimmune encephalitis could be evaluated or diagnosed by multiple methods for concluding the possibility or probability in patients with diverse AE syndromes independent from the serum or CSF antibody. However, these methods could not be applied in the retrospective study. And also, even though we could apply the scale for the AE diagnosis in the RCT research, at this time, we could not clearly classify them to each specific subtype, which might bring limitations in clearly describing clinical characteristics, immunotherapy regimens, and outcomes for each subtype of autoimmune encephalitis. In addition, theoretically speaking, AE associated with antibodies of GABAa Receptor, mGluR5, AMPA Receptors should also be included in this study, but the numbers of cases with these subtypes of AE were too limited (<10 cases) in the cohort to perform convincing analysis. Thus, we were not able to provide more detailed information here. All these cases were classified into others group, which might bring bias into this study.

This study describes multiple subtypes of autoimmune encephalitis clinical characteristics, immunotherapy regimens, and long-term outcomes of patients in China.

## Data Availability Statement

The original contributions generated in the study are included in the article/Supplementary Material, further inquiries can be directed to the corresponding author.

## Ethics Statement

The studies involving human participants were reviewed and approved by Beijing Tiantan Hospital Ethics Committee. Written informed consent to participate in this study was provided by the participants' legal guardian/next of kin.

## Author Contributions

WS collected the data and draft the manuscript. HY collected the data and analyzed part of the data. QW rechecked the data and revised the mansucript. All authors contributed to the article and approved the submitted version.

## Conflict of Interest

The authors declare that the research was conducted in the absence of any commercial or financial relationships that could be construed as a potential conflict of interest.

## Abbreviations

AEDs: antiepileptic drugs
AE: autoimmune encephalitis
IQR: interquartile range
NMDAR: anti-N-methyl-D-aspartate receptor
LGI-1: leucine-rich glioma-inactivated 1
GABAbR: γ-aminobutyric acid type B receptor
CASPR2: contactin-associated protein-2
MTX: methotrexate
RTX: rituximab
IVIG: IV immunoglobulin
PE: plasmapheresis
CTX: cyclophosphamide
MMF: mycophenolate mofetil
AZA: azathioprine.
