# Supplementary material for: Neuronal Surface Antibody-Medicated Autoimmune Encephalitis (Limbic Encephalitis) in China: A Multiple-Center, Retrospective Study
Source: Front Immunol. 2021 Feb 17;12:621599. doi: 10.3389/fimmu.2021.621599 (PMC7928315; doi:10.3389/fimmu.2021.621599)
Supplement: Supplementary file 1 [file Table_1.docx]

| **Supplementary Table 1 Demographic information of patients with autoimmune encephalitis** | | | | | | | | |
| --- | --- | --- | --- | --- | --- | --- | --- | --- |
|  | All (n=778) | NMDAR-AE (n=494), 63.50% | LGI-1 AE (n=160) 20.57% | GABAbR-AE (n=83) 10.67% | CRASPR-AE (n=26) 3.34% | Others (n=15) 1.93% |  |  |
| Median age, Range (y) | 35 [1-87] | 25 [1-87] | 61 [15-82] | 57 [17-82] | 43 [17-79] | 30 [10-66] |  |  |
| U18 (n, %) | 138, 17.74% | 127, 25.71% | 2, 1.25% | 2, 2.41% | 4, 15.38% | 3, 20.00% |  |  |
| Female | 354 (45.50%) | 252 (51.01%) | 54 (33.75%) | 31 (37.35%) | 9 (34.62%) | 8 (26.66%) |  |  |

| **Supplementary Table 2 Gender distribution of patients with autoimmune encephalitis** | | |
| --- | --- | --- |
|  | Gender (ratio) | Median age, Range (y) |
| NMDAR-AE | Female (50.39%) | 25, [1-87] |
|  | Male (49.61%) | 28, [2-82] |
| LGI-1-AE | Female (33.75%) | 58, [23-78] |
|  | Male (66.25%) | 61, [15-82] |
| GABAbR-AE | Female (37.35%) | 56, [17-84] |
|  | Male (62.65%) | 57, [28-82] |
| CRASPR-AE | Female (34.62%) | 40, [22-79] |
|  | Male (65.38%) | 44, [17-70] |
| Others-AE | Female (26.66%) | 35, [27-66] |
|  | Male (73.34%) | 26, [10-36] |

**Supplementary Table 3 CSF profile of patients with autoimmune encephalitis**

| **On-set** | NMDAR-AE | LGI-1 AE | GABAbR-AE | CRASPR-AE and Others |  |
| --- | --- | --- | --- | --- | --- |
| white blood cell count per ul | 39.26 (n=387) | 7.79 (n=134) | 29.93 (n=76) | N/A |  |
| protein concentration, mg/dL | 0.47 (n=297) | 0.553 (n=54) | 0.64 (n=43) | N/A |  |

| **Supplementary Table 4 EEG and MRI at diagnosis of patients with autoimmune encephalitis** | | | | | | | |  |
| --- | --- | --- | --- | --- | --- | --- | --- | --- |
| **On-set** | NMDAR-AE | LGI-1 AE | GABAbR-AE | CRASPR-AE and others | |  |  | |
| EEG abnormal (n), Ratio | 247/312, 79.16% | 76/126, 61.29% | 37/58 63.79% | 26/29 89.66% |  |  |  | |
| MRI abnormal (n), Ratio | 115/253 45.45% | 115/140, 82.14% | 61/81 75.31% | 33/36 91.67% |  |  |  | |
| Tumor (n) | 24/286 8.39% | 3/99, 3.03% | 10/64 15.63% | 2/38 5.26% |  |  |  | |

EEG abnormal including: abnormal sharp waves (epileptic discharge) and slow waves (1:11). MRI abnormal in the brain, mainly in the medial temporal lobe (about 36.44% in NMDAR-AE, 46.08% in LGI-1 AE, 50.00% in GABAbR-AE, 61.54% in CRASPR-AE and others). In the tumor group, NMDAR-AE 20 patients has been diagnosis with tumor 18/24 is ovarian teratoma, GABAbR-AE 9/10 is lung cancer.
